# Objective classification of deep squat and hurdle step scores in functional movement screen: a 3D motion capture and machine learning approach

**DOI:** 10.3389/fbioe.2026.1898662

**Published:** 2026-09-22

**Authors:** Zhenyang Xu, Zhiyuan Yang, Can Bu, Dewei Mao, Zhiyuan Sun, Qinghui Shang

**Affiliations:** 1 Institute of Sports Science, Shandong Sport University, Jinan, China; 2 Tianjin University of Sport, Tianjin, China; 3 Southeast University, Nanjing, China; 4 Faculty of Arts and Social Sciences—The Chinese University of Hong Kong, Shenzhen, China

**Keywords:** 3D motion capture, functional movement screen, machine learning, movement scoring, sports biomechanics

## Abstract

**Objective:**

Using 3D motion capture, inverse dynamics analysis, and machine learning methods, this study investigates the biomechanical differences across scoring grades of the deep squat (DS) and hurdle step (HS) tasks in the Functional Movement Screen (FMS), and evaluates the feasibility of objectively classifying FMS scores.

**Methods:**

The study included 100 healthy adolescents, all of whom completed the DS and HS tasks. For the DS, 100 valid movement trials were obtained; for the HS, which included hurdle steps on both sides, a total of 200 valid unilateral trials were obtained. A 12-camera infrared optical motion capture system was used to simultaneously collect kinematic data, which was combined with ground reaction forces for inverse dynamics analysis to extract features, including joint angles, joint moments, range of motion (ROM), and bilateral differences. The DS data were analyzed at the trial level, while the HS data were aggregated at the subject level after accounting for bilateral correlations. The Kruskal-Wallis H test and Mann-Whitney U test were used to compare differences across scoring grades. Cross-validation was performed based on subject IDs to construct logistic regression, support vector machine (SVM), random forest, and Ordinal Random Forest (OrdinalRF) models.

**Results:**

In the DS task, significant differences were observed across scoring levels for the ankle dorsiflexion angle difference, ROM of pelvic vertical displacement, and trunk flexion ROM. In the HS task, the hip flexion difference and pelvic rotation ROM demonstrated strong discriminatory power across scoring levels. The Random Forest model performed well in the DS task, with an accuracy of 0.910 ± 0.080 and a squared-weighted Kappa of 0.904 ± 0.061; in the HS task, OrdinalRF showed the highest descriptive performance, with an accuracy of 0.805 ± 0.053 and a squared-weighted Kappa of 0.728 ± 0.094. Misclassifications primarily occurred between adjacent rating levels.

**Conclusion:**

The FMS scores for the DS and HS tasks are based on different biomechanical criteria. The combination of 3D motion capture with inverse dynamics analysis and machine learning provides quantitative support for the objectification of FMS scores; however, feature overlap and classification uncertainty still exist between adjacent grades.

## 1 Introduction

The Functional Movement Screen (FMS) is widely used in sports training and rehabilitation assessment (Bonazza et al., 2017; Moran et al., 2016), providing a comprehensive analysis of an individual’s range of motion (ROM), stability, and symmetry (Cook et al., 2006; Cook et al., 2014). The deep squat (DS), as a typical bilateral closed-chain movement, primarily reflects the lower extremities’ multi-joint coordination and dynamic stability (Cook et al., 2014; Kritz et al., 2009; Wan et al., 2021; Yuan and Yehuan, 2026; Hao et al., 2024); the hurdle step (HS), on the other hand, is a typical unilateral dynamic task that emphasizes single-leg stability and the coordinated control of the pelvis and hip joints (Cook et al., 2014; Yuan and Yehuan, 2026; Zhang and Li, 2025). Consequently, both tasks are widely used to identify movement pattern deficits and assess functional movement quality. (Kiesel et al., 2007). FMS scores are primarily based on the evaluator’s subjective observation and judgment of movement quality, and the results are influenced by the evaluator’s experience and understanding of the scoring criteria (Zhang and Yang, 2020).

Quantitative analysis of functional movements using objective biomechanical data can reduce the subjectivity of manual scoring and provide deeper insights into the biomechanical mechanisms underlying differences in movement scores (Remedios et al., 2020; Macmillan et al., 2023). Existing studies have primarily focused on analyzing sagittal plane kinematic characteristics in the DS, such as trunk flexion and hip, knee, and ankle flexion, to explain the kinematic basis for scoring differences (Macmillan et al., 2023; Butler et al., 2010), while relatively little attention has been paid to the HS task, which relies more heavily on unilateral support and coordinated control of the pelvis and hip joint (Cook et al., 2014; Zhang and Li, 2025; Remedios et al., 2020). In particular, there is a lack of systematic comparisons between the DS and HS tasks regarding differences in key biomechanical characteristics and their ability to distinguish between performance levels. Studies have shown that the use of depth cameras, multi-view skeleton data, and deep learning models for the automatic assessment of FMS movements has yielded good results in 0–3 or 1–3 point scoring prediction tasks (Shen et al., 2024; Xing et al., 2022; Hong et al., 2023; Spil et al., 2022). However, current research has primarily focused on optimizing sensor configurations and model performance, relying mainly on skeleton sequences or video data, with limited integration of high-precision 3D motion capture data and inverse kinematic parameters. Additionally, explanations for the model’s decision-making criteria remain relatively limited, and there has been a lack of in-depth exploration into the core question of which key biomechanical features drive the differences between different scoring levels.

Therefore, this study employs 3D motion capture, inverse dynamics analysis, and machine learning to comprehensively analyze the key biomechanical characteristics of the DS and HS movements in the FMS, as well as their scoring and classification capabilities. A 1–3 point classification model was constructed to compare the two tasks in terms of both classification performance and feature contributions. The findings aim to improve the objectivity of FMS scoring and offer guidance for refining functional movement assessment protocols for different tasks. The present study focuses specifically on the objective characterization and classification of FMS scores and does not evaluate injury risk or injury-related outcomes.

## 2 Materials and Methods

Participants underwent FMS assessment while 3D motion capture data were collected. Biomechanical features, including joint angles and torques, were extracted using inverse kinematics and inverse dynamics. Peak values, ROM, and bilateral differences were then calculated; Nonparametric statistical analysis was used to compare feature across scoring levels. Machine learning models were constructed based on selected key features, and their classification performance was evaluated using cross-validation. Model results were interpreted in conjunction with feature importance (Figure 1).

**FIGURE 1:**
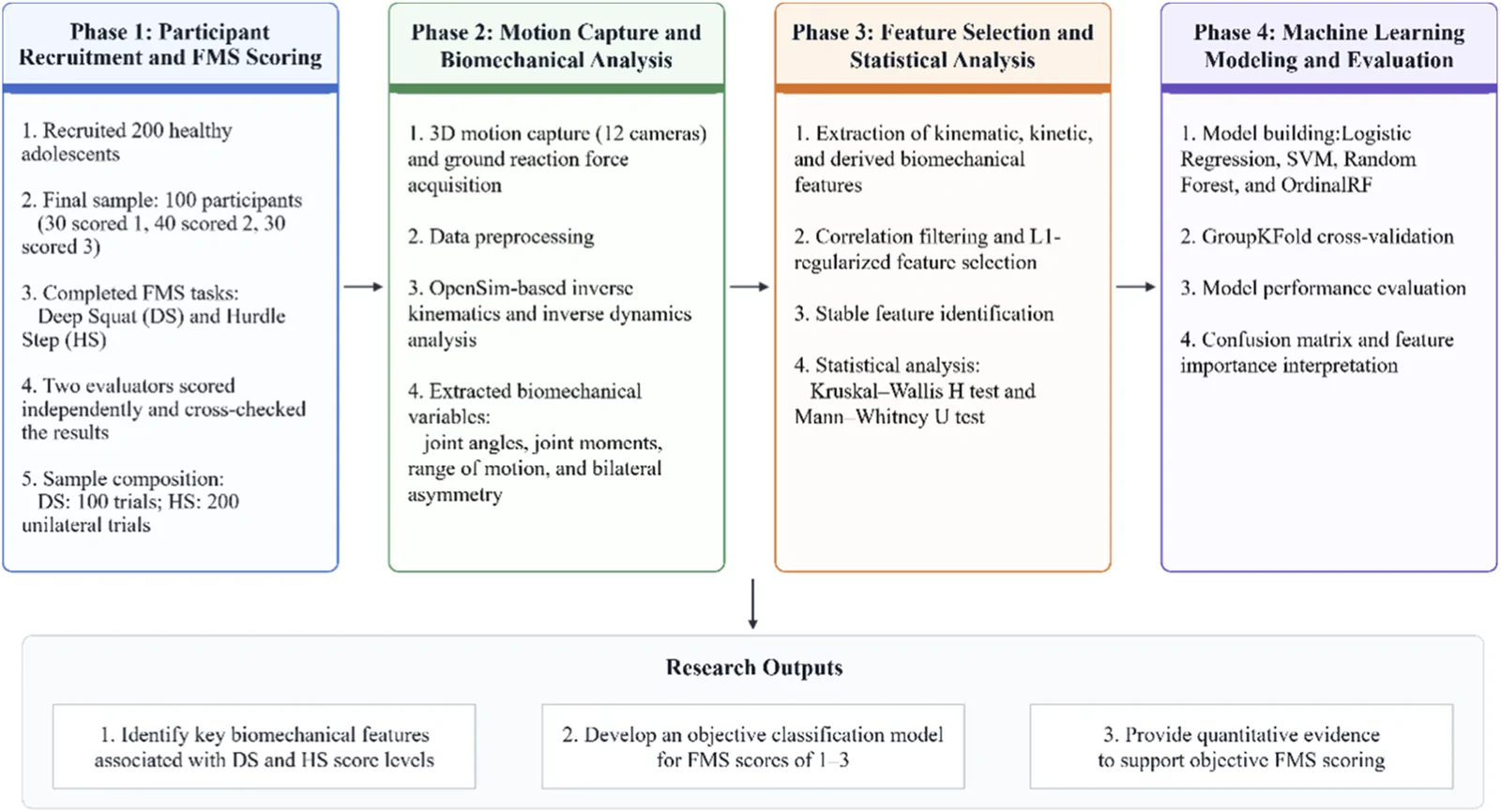
Study flowchart.

### 2.1 Study population

Following previous studies (Remedios et al., 2020), this study employed a stratified purposive sampling strategy to ensure relatively balanced sample sizes across FMS score categories for machine learning modeling. Therefore, this study focuses on exploring biomechanical differences across score categories and the feasibility of model classification, rather than estimating the true distribution of FMS scores categories in the general population of healthy adolescents. A preliminary sample of 200 healthy adolescents, all enrolled in physical education colleges, completed the Deep Squat (DS) and Hurdle Step (HS) tasks and received FMS scores from certified evaluators. Because the natural distribution of FMS scores was potentially imbalanced, we adopted a stratified purposive sampling strategy to obtain a relatively balanced sample across DS score categories for subsequent biomechanical comparison and machine learning analysis. Participants were first stratified according to their DS scores (1, 2, or 3). Within each score category, participants were randomly selected until the prespecified analytical sample sizes were reached (30, 40, and 30 participants for scores 1, 2, and 3, respectively). No additional demographic or anthropometric characteristics, including age, sex, height, weight, or BMI, were used to determine participant selection.

The final sample consisted of 100 participants, with 30, 40, and 30 participants classified as Level 1, 2, and 3 in the DS task, respectively. To minimize participant fatigue and maintain consistency with the official FMS assessment procedure, each movement was scored based on a single valid trial after standardized instruction and familiarization. The purpose of this study was to characterize biomechanical differences among FMS score categories rather than to estimate within-subject movement variability across repeated trials. Trials were repeated when participants failed to complete the movement according to the standardized protocol or when technical issues occurred, such as marker loss, incomplete movement execution, or unsuccessful biomechanical processing. Only trials that met all quality-control criteria were retained for subsequent scoring and analysis. During data collection, 3 DS trials and 6 HS trials required repetition because the initial attempts did not meet the predefined movement-execution or data-quality criteria. These 9 invalid trial attempts were excluded and replaced by valid repeat trials. Consequently, the final analytical dataset comprised 100 valid DS trials and 200 valid unilateral HS trials. Each participant performed one DS trial, yielding 100 valid DS trials, including 30 Level 1 trials, 40 Level 2 trials, and 30 Level 3 trials. For the HS task, each participant performed one trial on each side, resulting in 200 valid unilateral HS trials. For machine learning analysis, these unilateral HS trials were retained to fully use side-specific movement information, and subject-ID-based group cross-validation was applied to prevent trials from the same participant from appearing simultaneously in the training and testing sets. However, for between-group statistical comparisons of the HS task, left- and right-side trials from the same participant were not treated as independent observations due to within-subject correlation. Instead, HS data were aggregated at the participant level. Continuous biomechanical variables were averaged across the left and right sides, and the lower score of the two sides was used as the participant-level HS score according to the FMS unilateral scoring principle. After aggregation, the HS dataset for statistical analysis included 100 participants, comprising 52 with a score of 1, 35 with a score of 2, and 13 with a score of 3. It should be noted that the final sample was selected to improve score-level balance for analytical purposes and therefore does not represent the natural distribution of FMS scores among healthy adolescents.

This study was approved by the Ethics Committee of Shandong Sport University (Ethics No.: 2025077). Inclusion criteria: healthy adolescents aged 14–18 years; voluntary participation and signing of an informed consent form; ability to independently perform the FMS squat and hurdle step movements. Participants who did not meet any of the above criteria were excluded. Table 1 presents demographic information and baseline characteristics of participants across different FMS score categories. There were no statistically significant differences among the three groups in terms of age, gender, height, weight, or BMI (*p* > 0.05), suggesting good baseline comparability across the score categories.

**TABLE 1:** Comparison of baseline characteristics among participants with different FMS score categories.

Variables	Group 1 (N = 30)	Group 2 (N = 40)	Group 3 (N = 30)	*p*
Age (years)	17.2 ± 1.0	17.4 ± 1.0	17.0 ± 1.2	0.344
Gender	Man: 18 (60%) Woman: 12 (40%)	Man: 20 (50%) Woman: 20 (50%)	Man: 12 (40%) Woman: 18 (60%)	0.301
Height (cm)	169.9 ± 7.5	171.7 ± 7.3	171.4 ± 8.3	0.587
Weight (kg)	58.2 ± 9.4	60.7 ± 10.1	61.8 ± 12.9	0.436
BMI (kg/m^2^)	20.1 ± 2.2	20.5 ± 2.5	20.8 ± 2.8	0.524

### 2.2 Experimental tasks and grading criteria

Participants first received a standardized explanation of the experimental procedure and performed familiarization exercises prior to the formal data collection for each movement (Figure 2). Formal scoring and data analysis were based on a single valid trial. If obvious marker loss, incomplete movement execution, or failure to meet test requirements occurred during data collection, the trial was repeated or excluded. As described by Cook et al. (2014), the FMS testing criteria employ a 1–3 point rating scale. Each participant sequentially performed the DS and HS tasks, with scoring conducted independently by two assessors who had completed formal FMS certification training and had doctoral-level professional backgrounds in sports rehabilitation and related disciplines. Both assessors had prior practical experience in administering and scoring the FMS. Before formal scoring, they jointly reviewed the standardized FMS scoring criteria for the DS and HS tasks and practiced scoring sample movement trials. Any discrepancies identified during the calibration process were discussed to establish a consistent interpretation of the scoring criteria. Inter-rater agreement was assessed using quadratic-weighted Cohen’s kappa, reflecting the ordinal nature of the 1–3 FMS scores and assigning smaller penalties to disagreements between adjacent score categories than to disagreements spanning two score levels. A kappa value of ≥0.60 was prespecified as the minimum acceptable level of agreement based on the commonly used Landis and Koch interpretation framework (Landis and Koch, 1977). If this threshold had not been reached, the assessors would have undergone additional calibration by jointly reviewing the FMS scoring criteria and discrepant movement videos, followed by repeat scoring before the labels were accepted for subsequent analysis. When the two assessors’ scores were inconsistent, they jointly reviewed the movement videos and the FMS scoring criteria to determine the final score, which was used as the label for the machine learning model. It should be noted that this threshold primarily ensures an acceptable level of consistency and does not imply that the scoring is completely stable or error-free. For the DS task, each participant performed one squat. The HS task included two directions: left-side and right-side hurdle step. During the statistical analysis phase, the correlation between left- and right-side movements of the same participant was considered, and data were aggregated at the participant level. Continuous biomechanical variables were averaged across the left and right sides, and the lower of the two FMS scores was used as the participant-level HS score. For machine learning modeling, the 200 unilateral trials derived from the 100 participants were retained as separate observations, and cross-validation was performed by grouping based on subject IDs to prevent data leakage.

**FIGURE 2:**
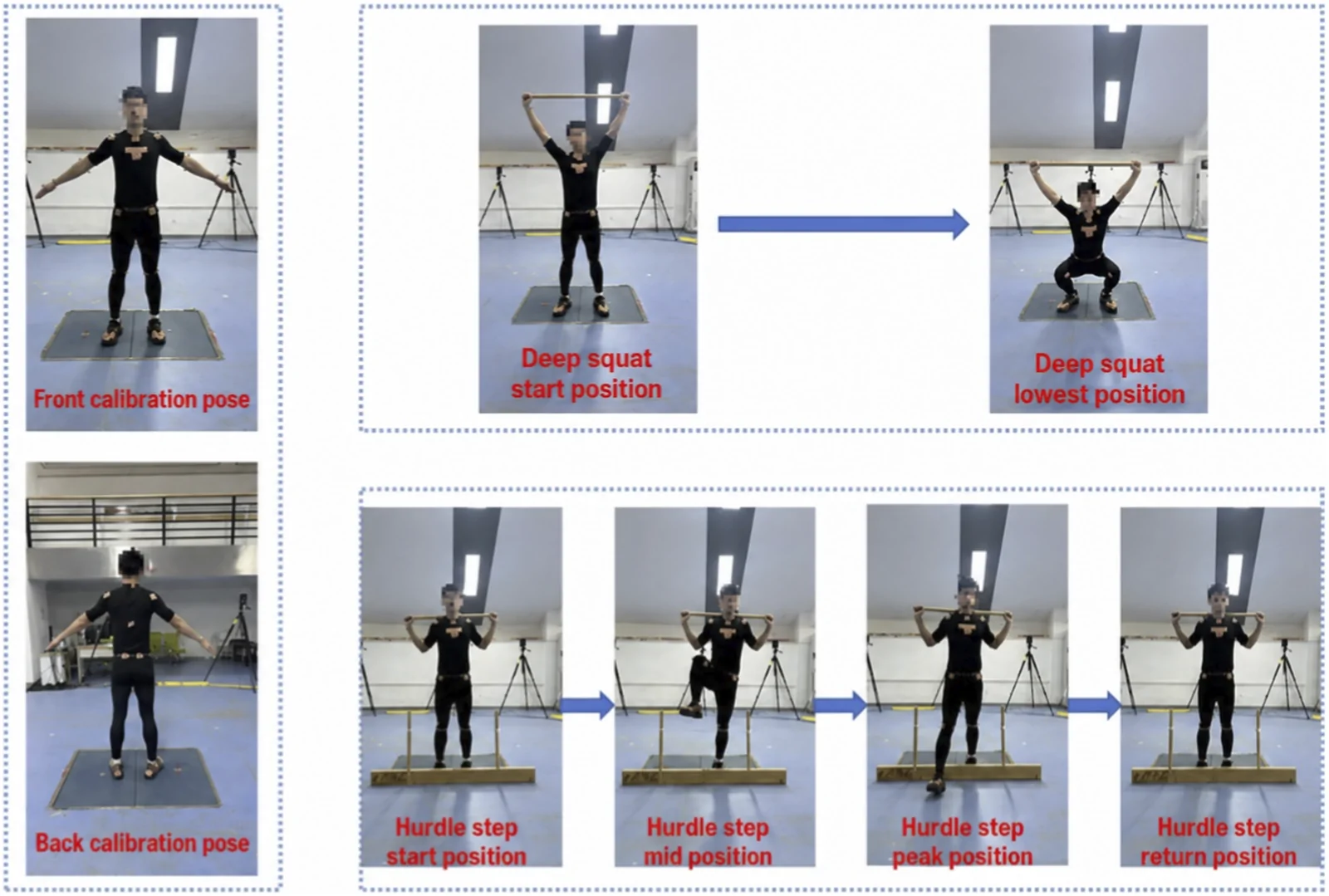
Experimental setup and schematic diagrams of the squat and hurdle stride movements.

Therefore, in this study, the sample unit for the DS task is a single squat movement trial. For the HS task, statistical analysis is aggregated at the participant level, while machine learning modeling is conducted at the level of single-sided hurdle step movement trials. These two analytical scales were used for different purposes: participant-level aggregation was used for inferential comparisons while accounting for bilateral dependence, whereas unilateral trials were retained for machine learning to preserve side-specific information. Accordingly, statistical between-group differences and model-based feature importance for the HS task should be interpreted as complementary rather than directly equivalent evidence.

### 2.3 Motion capture and kinematic calculations

A 12-camera infrared optical motion capture system (Nokov, China) was used to collect three-dimensional kinematic data at a sampling rate of 200 Hz, recording the three-dimensional coordinates of 36 reflective markers. An AMTI three-dimensional force plate (Advanced Mechanical Technology Inc., United States) was used for static calibration and dynamic data collection at a sampling rate of 1 kHz, synchronized in time with the motion capture system. Both kinematic and ground-reaction-force data were low-pass filtered using a fourth-order zero-phase Butterworth filter with a cutoff frequency of 6 Hz. The 6-Hz cutoff was selected to reduce high-frequency measurement noise while preserving the low-frequency movement characteristics of the DS and HS tasks. No additional resampling of the ground-reaction-force data was performed within the OpenSim inverse-dynamics workflow.

The motion-capture data were processed in OpenSim 4.0 (SimTK, United States) using the Rajagopal full-body musculoskeletal model (Rajagopal et al., 2016). A customized 36-marker set was mapped to the corresponding anatomical landmarks of the generic model. The anatomical locations of all 36 experimental markers and their correspondence to the customized virtual marker set of the OpenSim model are provided in Supplementary Table S1. The original joint coordinate definitions and degrees of freedom of the musculoskeletal model were retained, while the marker set was customized to match the experimental 36-marker protocol. For each participant, the model was individually scaled using anthropometric measurements and marker positions obtained from a static calibration trial. Segment inertial properties were inherited from the generic musculoskeletal model and adjusted during participant-specific scaling. The model coordinates relevant to the present analysis included pelvic tilt, pelvic list, and pelvic rotation; bilateral hip flexion–extension, hip adduction–abduction, and hip rotation; knee flexion–extension; ankle dorsiflexion–plantarflexion; and trunk/lumbar flexion–extension, lateral bending, and axial rotation. Joint coordinate definitions and positive directions followed those implemented in the Rajagopal model. Joint angles were calculated using inverse kinematics. Inverse-kinematics tracking quality was considered acceptable when the RMS marker error was <0.02 m and the maximum marker error was <0.04 m. Inverse dynamics was subsequently performed using the participant-specific scaled model, the inverse-kinematics-derived joint coordinates, and the synchronized ground reaction force data. The measured external loads were incorporated into the inverse-dynamics analysis using the OpenSim external-load framework. The joint moments used in the subsequent analyses corresponded to the sagittal-plane generalized coordinates of the lower extremity, including hip flexion–extension, knee flexion–extension, and ankle dorsiflexion–plantarflexion moments. Joint moments were normalized by body mass (Nm/kg). For selected peak-moment features, absolute values were used where appropriate to represent moment magnitude irrespective of sign.

Missing kinematic values were handled using linear interpolation, with remaining boundary missing values filled by backward or forward filling. No explicit maximum gap-length threshold was prespecified; trials with substantial marker loss or incomplete movement data that could not be validly processed were excluded. A Hampel filter was subsequently applied for outlier removal. The processed marker trajectories were used for inverse kinematics, while the synchronized ground-reaction-force data were incorporated into OpenSim through trial-specific External Loads files for inverse-dynamics analysis. External forces were assigned to the corresponding right and left calcaneus segments (calcn_r and calcn_l) and expressed in the ground coordinate system. Subsequently, the inverse-kinematics and inverse-dynamics time series used for feature extraction were time-normalized to 101 points, representing 0%–100% of the movement cycle. For key moment identification, the lowest squat position in the DS task was defined as the moment when the average time series of left and right knee flexion angles reached its maximum. In the HS task, the highest point of the hurdle jump was defined as the moment when the hip flexion angle on the stride side reached its maximum. The above processing procedures were applied consistently across all participants and movement trials.

### 2.4 Feature extraction and selection

Based on 3D motion capture data and inverse kinematic analysis results, this study extracted kinematic, dynamic, and derived biomechanical features for both the DS and HS tasks. For the DS task, the extracted features included lower limb joint angles, trunk posture, pelvic control, bilateral symmetry, and joint torques. For the HS task, the features included joint angles of the hurdling leg and supporting leg, trunk and pelvic control, lumbar spine ROM, bilateral differences, and joint torques. Pelvic Rotation ROM and Lumbar Spine Rotation ROM were treated as distinct variables, representing axial rotation of the pelvis and lumbar spine, respectively. To facilitate presentation and interpretation, Table 2 lists the representative features most closely related to task characteristics and model discrimination. The complete set of extracted features is provided in Supplementary Table S2. To reduce feature redundancy and mitigate overfitting under small-sample conditions, a two-stage feature selection process was nested within cross-validation. First, within each training fold, the Spearman correlation coefficient matrix among candidate features was calculated. Features with an absolute correlation coefficient |r| > 0.85 were identified as highly correlated and removed to eliminate redundancy. This step aimed to reduce redundancy among features rather than to conduct a hypothesis test for the significance of correlations; therefore, no correction for multiple comparisons was performed. The threshold 0.85 was chosen based on common practice in machine learning for removing highly correlated features, as well as the small number of initial features and the limited sample size in this study. Subsequently, the training set features were z-score standardized, and L1-regularized logistic regression was employed for further feature selection. The L1 model utilized the SAGA solver with a maximum of 5,000 iterations, class weights set to “balanced,” and a regularization parameter C set to 1.0. Features with non-zero coefficients that exceeded the model selection threshold were considered candidate contributing features for the current training fold. All correlation screening, standardization, and L1 feature selection were performed exclusively on the training set, and the processing workflow determined on the training set was applied to the corresponding test set to avoid data leakage.

**TABLE 2:** List of representative features for DS and HS tasks.

Task	Characteristic	Definition	Calculation method
DS	Ankle dorsiflexion angle difference	Ankle dorsiflexion angle difference s between the left and right sides	Absolute value of the difference between the peak ankle dorsiflexion angles on the left and right sides
	ROM of pelvic vertical displacement	The range of vertical displacement of the pelvis during a squat, reflecting the depth of the squat	Maximum value minus minimum value
	Trunk flexion ROM	Range of trunk flexion during a squat	Maximum − minimum
HS	Hip flexion difference	Hip flexion difference angles between the stride leg and the support leg	Absolute value of the difference between the peak hip flexion angles on both sides
	Lumbar spine lateral flexion ROM	Range of lumbar lateral flexion angles during the hurdle stride	Maximum value − minimum value
​	Lumbar spine rotation ROM	Range of lumbar spine axial rotation during the hurdle stride	Maximum value − minimum value

Abbreviations: ROM, range of motion; DS, deep squat; HS, hurdle step.

Subject-based five-fold cross-validation was used to calculate the selection frequency of each feature across the five folds. A 100% selection frequency was used as the criterion for determining final stable features in the primary analysis, while sensitivity analyses were conducted using thresholds of ≥60% and ≥80%. Given the five-fold design, ≥60%, ≥80%, and 100% correspond to selection in at least 3, 4, and 5 folds, respectively. Feature retention under these three thresholds was compared. The entire feature engineering process was strictly nested within the cross-validation framework, using only training set data to ensure an unbiased estimate of the generalization error.

### 2.5 Machine learning models and evaluation methods

Logistic regression, support vector machines (SVM), and random forests (RF) were used to build three-class (1–3) classification model for FMS scores. These three models represent linear interpretable, nonlinear kernel, and ensemble tree models, respectively, allowing evaluation of how kinematic and dynamic features discriminates FMS scores under different decision boundaries and complexity levels. Given the small sample size of this study and the primary objective of establishing an interpretable, objective scoring model, the aforementioned traditional machine learning models were prioritized, while models with higher sample size requirements, such as deep neural networks, were excluded. Although gradient boosting models like XGBoost possess strong predictive capabilities, they are more prone to overfitting under conditions of small sample sizes and multiple cross-validation, and thus were not selected as primary models. Given the inherent ordinal nature of FMS scores (1, 2, 3), an OrdinalRF model was additionally constructed for supplementary analysis. All machine-learning analyses were implemented in Python using scikit-learn version 1.8.0 and imbalanced-learn version 0.14.1. A fixed random state of 42 was used throughout the analysis for reproducibility. Logistic Regression used the multinomial formulation with the L-BFGS solver and a maximum of 5,000 iterations, whereas the L1-regularized Logistic Regression used for feature selection employed the SAGA solver. The SVM used a radial basis function (RBF) kernel. Random oversampling was implemented using RandomOverSampler with random_state = 42 and the default sampling_strategy = “auto”, whereby minority classes within each training partition were randomly oversampled with replacement to match the size of the majority class. The resulting class distributions before and after oversampling across the five outer-training folds are summarized in Supplementary Table S3. Oversampling was restricted to the training partitions within the cross-validation pipeline.

OrdinalRF was custom implemented rather than obtained from an existing ordinal-classification package. The three-level outcome was decomposed into two binary classification problems corresponding to y > 1 and y > 2, each fitted using a Random Forest classifier. For prediction, the positive-class probability from each binary classifier was thresholded at 0.50, and the final FMS score was calculated as 1 + I [P (y > 1) ≥ 0.50] + I [P (y > 2) ≥ 0.50]. Thus, the two binary outputs were combined to yield a final score of 1, 2, or 3. If the two independently fitted classifiers produced an inconsistent threshold pattern, a single positive threshold prediction resulted in an intermediate score of 2. Class weighting was used at different stages of the modeling pipeline. The L1-regularized feature-selection step preceded random oversampling and used the “balanced” class-weight strategy to account for the original class imbalance during feature selection. For the standard three-class classifiers, random oversampling resulted in equal class frequencies before classifier fitting; consequently, the subsequent balanced class weights were equal and did not further modify the relative contributions of the three score categories. For the custom OrdinalRF, class weighting remained relevant because the balanced three-class outcome was subsequently decomposed into two binary threshold problems (y > 1 and y > 2), within which class imbalance could re-emerge.

Model evaluation used nested, subject-based cross-validation. Random oversampling, standardization, feature selection, and model training were encapsulated within the cross-validation pipeline. All data-dependent operations were performed exclusively on the corresponding training partitions and then applied to the validation or test data without refitting. The outer layer employed 5-fold GroupKFold to assess generalization performance, ensuring that all trials from the same subject do not appear in both the training and test sets. The inner layer used GroupKFold for hyperparameter grid search on the training set typically set to 3-fold; when the number of available subject groups in the training set is less than 3, it is adjusted according to the actual number of available groups. The grid search uses balanced accuracy as the optimization objective. For Logistic Regression, the regularization parameter C uses logarithmic-scale candidate values of 0.01, 0.1, 1, and 10; for SVM, C is set to 0.1, 1, and 10, and gamma is set to scale, 0.1, and 0.01; for Random Forest and OrdinalRF, the number of trees is set to 200 or 300, the maximum depth is set to None, 3, 5, or 8, and the minimum split sample size is set to 2, 5, or 10.

Model performance was evaluated using both conventional classification metrics and ordinal performance metrics. Balanced accuracy was prespecified as the primary metric for hyperparameter optimization and model comparison because it gives equal importance to each score category and is less affected by class imbalance than conventional accuracy. Accordingly, the best-performing model for each task was defined as the model with the highest mean balanced accuracy across the five outer-test folds. Accuracy, macro-average F1 score, and weighted F1 score were reported as complementary conventional classification metrics. Given the ordinal nature of FMS scores, MAE and quadratic weighted kappa (QWK) were additionally reported as complementary ordinal metrics. QWK accounts for the ordered nature and magnitude of disagreements between score categories, whereas MAE directly quantifies the average absolute scoring error. These ordinal metrics were therefore used to characterize the severity of classification errors but did not override balanced accuracy as the primary model-selection criterion. All performance metrics are reported as the mean and standard deviation across the five outer-test folds. Model performance evaluation and feature interpretation were treated as separate processes. Confusion matrices were presented for the model with the highest mean outer-fold balanced accuracy for each task to illustrate classification patterns across scoring levels. The confusion matrices were constructed by aggregating the out-of-fold predictions from all five outer test folds. Each observation therefore contributed one prediction obtained when it was held out in an outer test fold. No separately refitted final model was used to generate the confusion matrices. Feature importance analysis was uniformly based on the Random Forest model to provide a consistent interpretation framework across the DS and HS tasks. Feature importance was quantified using the impurity-based importance implemented by the Random Forest classifier (mean decrease in impurity, MDI). Importance values were averaged across the outer cross-validation folds after mapping fold-specific selected features back to the full candidate-feature space, with features not selected in a given fold assigned an importance of zero. These values reflect the relative predictive relevance of biomechanical features within the fitted Random Forest models and should not be interpreted as evidence of causal biomechanical effects. Feature importance was used only for model interpretation and did not determine the selection of the best-performing classifier.

### 2.6 Statistical analysis methods

Statistical analyses were performed using Python 3.9. Continuous variables were expressed as mean ± standard deviation or median (IQR). Normality and homoscedasticity were tested using Shapiro–Wilk and Levene’s tests (Supplementary Table S4). The results showed that most biomechanical features in both the DS and HS tasks did not follow a normal distribution in at least one scoring category, and some features exhibited heteroscedasticity. Given that most features did not meet the assumptions for parametric tests and that the FMS score is an ordinal variable, this study employed nonparametric methods to analyze differences between scoring categories. For the HS task, between-group statistical comparisons were conducted at the participant level as described in Section 2.2. The resulting participant-level sample comprised 100 participants: 52 with a score of 1, 35 with a score of 2, and 13 with a score of 3.

The Kruskal-Wallis H test was used to compare overall differences among the groups with scores of 1, 2, and 3. For variables showing statistically significant overall differences, pairwise comparisons were further performed using the Mann-Whitney U test, with multiple comparisons corrected using the Holm-Bonferroni method. Effect size measures included the ε^2^ from the Kruskal-Wallis H test and the two-way rank correlation coefficients from the pairwise comparisons. The performance of machine learning models was evaluated using outer 5-fold GroupKFold cross-validation and reported as mean ± standard deviation. Since the folds in the cross-validation were not completely independent and the sample size in this study was limited, no significance tests were performed between the performance of different models; only descriptive comparisons were conducted. All statistical tests were two-sided, with a significance level set at *p* < 0.05.

## 3 Result

### 3.1 Dataset

A total of 100 participants were ultimately included. For the DS task, 100 valid movement trials were obtained, comprising 30 score-1, 40 score-2, and 30 score-3 trials. For the HS task, each participant contributed one left- and one right-sided trial, yielding 200 unilateral movement trials from 100 participants for machine learning analysis, comprising 60 score-1, 80 score-2, and 60 score-3 trials. For participant-level statistical analysis, the aggregated HS sample comprised 100 participants: 52 with a score of 1, 35 with a score of 2, and 13 with a score of 3.

The weighted Kappa coefficients for the DS and HS tasks were 0.90 and 0.66, respectively. According to the interpretation criteria proposed by Landis JR and Koch (Landis and Koch, 1977), agreement for the DS task reached a near-perfect level, whereas agreement for the HS task was lower and remained at an acceptable level. This suggests that manual scoring for HS involves a higher degree of uncertainty.

### 3.2 Feature analysis

Overall, no feature pairs exceeded the predefined correlation threshold of |r| > 0.85 in either the DS or HS task, indicating that no features were removed because of excessive pairwise correlation. In the DS task (Figure 3A), the ROM of pelvic vertical displacement showed a moderate positive correlation with hip flexion angle (r = 0.49) and a weak-to-moderate positive correlation with peak knee joint torque (r = 0.30). Ankle dorsiflexion angle showed a moderate negative correlation with peak pelvic vertical position (r = −0.57) and trunk flexion angle (r = −0.50). Furthermore, the ankle dorsiflexion angle difference showed a strong negative correlation with the ROM of pelvic vertical displacement (r = −0.71). Finally, trunk flexion ROM showed a weak-to-moderate negative correlation with peak knee joint torque (r = −0.31).

**FIGURE 3:**
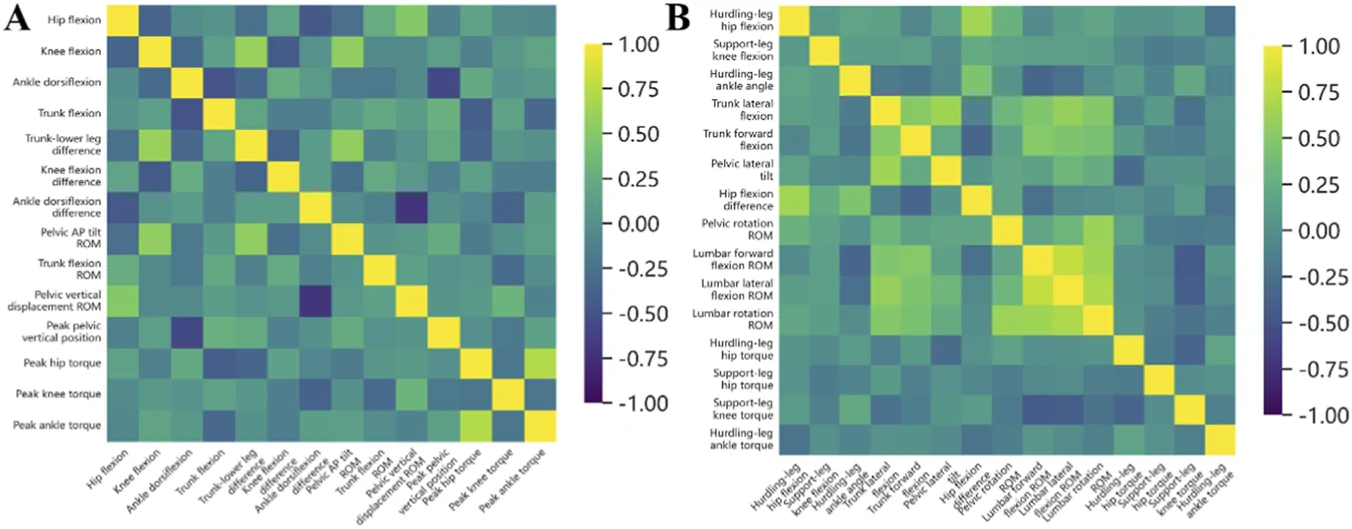
Heat maps showing the correlations among key biomechanical features across different movement tasks. **(A)** Heat map for the squat task; **(B)** Heat map for the hurdle step task.

In the HS task (Figure 3B), moderate to strong positive correlation were observed among lumbar spine forward flexion ROM, lumbar spine lateral flexion ROM, and lumbar spine rotation ROM. Specifically, the lumbar spine forward flexion ROM was highly correlated with lateral flexion ROM (r = 0.79), and the lumbar spine lateral flexion ROM was strongly correlated with that for rotation ROM (r = 0.71). Pelvic Rotation ROM showed a moderate positive correlation with Lumbar Spine Rotation ROM (r = 0.62) and a weak-to-moderate positive correlation with Lumbar Spine Lateral Flexion ROM (r = 0.32). Hip flexion difference was moderately correlated with hip flexion angle of the hurdling leg (r = 0.64) and with ankle angle of hurdling leg (r = 0.45).

Sensitivity analysis across feature-selection frequency thresholds of ≥60%, ≥80%, and 100% showed that the primary selected features were generally stable (Table 3). For the DS task, ankle dorsiflexion angle difference, pelvic vertical displacement ROM, and trunk flexion ROM were retained across all three thresholds. For the HS task, hip flexion difference, lumbar spine lateral flexion ROM, and pelvic rotation ROM were consistently retained at the ≥80% and 100% thresholds.

**TABLE 3:** Feature selection results for the DS and HS tasks under different selection frequency thresholds.

Task	Selection frequency threshold	Minimum selection ratio	Number of features to retain	Features to retain
DS	≥60%	≥3/5 fold	4	Ankle dorsiflexion angle difference, pelvic vertical displacement ROM, and trunk flexion ROM, trunk flexion angle
	≥80%	≥4/5 fold	4	Ankle dorsiflexion angle difference, pelvic vertical displacement ROM, and trunk flexion ROM, trunk flexion angle
	100%	5/5 fold	3	Ankle dorsiflexion angle difference, pelvic vertical displacement ROM, and trunk flexion ROM
HS	≥60%	≥3/5 fold	4	Hip flexion difference, lumbar spine lateral flexion ROM, and pelvic rotation ROM, lumbar spine rotation ROM
	≥80%	≥4/5 fold	3	Hip flexion difference, lumbar spine lateral flexion ROM, and pelvic rotation ROM
	100%	5/5 fold	3	Hip flexion difference, lumbar spine lateral flexion ROM, and pelvic rotation ROM

Since this study employs five-fold cross-validation, ≥60%, ≥80%, and 100% correspond to at least 3, 4, and 5 folds, respectively. Abbreviations: ROM, range of motion; DS, deep squat; HS, hurdle step.

As shown in Figure 4, key biomechanical characteristics exhibited different distribution patterns across FMS score levels in the DS and HS tasks. In the DS task, ankle dorsiflexion angle difference (H = 68.73, ε^2^ = 0.69), pelvic vertical displacement ROM (H = 66.40, ε^2^ = 0.66), and trunk flexion ROM (H = 38.58, ε^2^ = 0.38) all showed significant differences across scoring levels (*p* < 0.001). In the HS task, significant differences across scoring levels were observed for hip flexion difference (H = 32.74, ε^2^ = 0.32) and pelvic rotation ROM (H = 26.71, ε^2^ = 0.25) across different scoring levels (*p* < 0.001). In contrast, between-group differences in lumbar spine lateral flexion ROM did not reach statistical significance (H = 1.23, *p* = 0.541).

**FIGURE 4:**
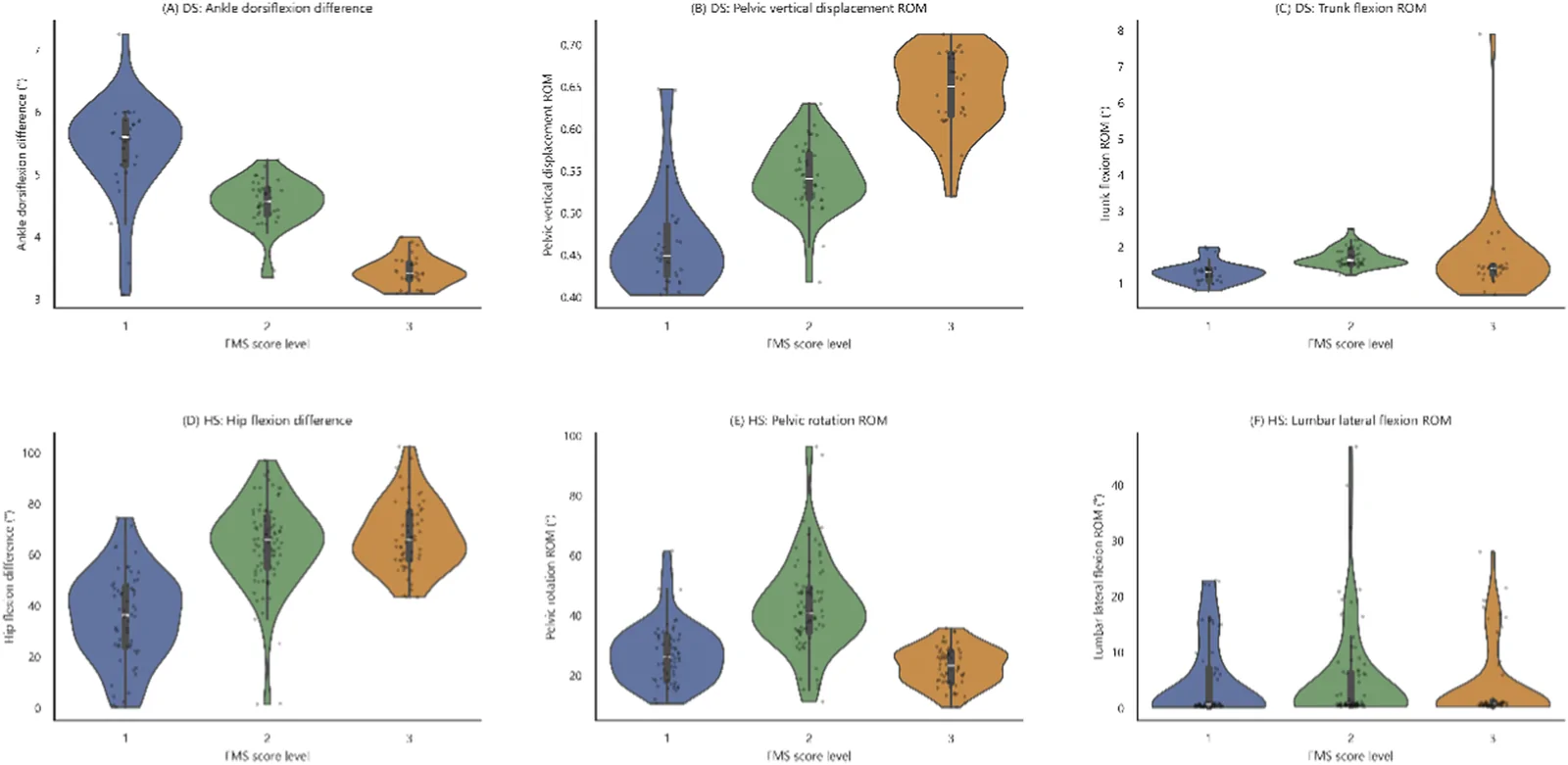
Distribution of key biomechanical characteristics for the DS and HS tasks across different FMS score levels. ROM, range of motion; DS, deep squat; HS, hurdle step.

Further pairwise comparisons were conducted for the key features (Table 4). In the DS task, significant differences in ankle dorsiflexion angle difference, pelvic vertical displacement ROM, and trunk flexion ROM were observed across most scoring levels. This indicates that changes in DS scores are closely related to ankle symmetry, pelvic vertical displacement, and trunk control ability. In the HS task, hip flexion difference was significant between Group 1 and Group 2, as well as between Group 1 and Group 3, but not between Group 2 and Group 3. This suggests that this feature primarily distinguishes low-score movements from higher-score movements. The Pelvic rotation ROM differed significantly between Group 1 and Group 3, as well as between Group 2 and Group 3, indicating that higher-scoring groups exhibit better pelvic rotation control. No significant differences were found among the three groups for lumbar spine lateral flexion ROM, indicating that although this feature is relatively stable in feature selection, its discriminatory power in univariate analysis is limited.

**TABLE 4:** Between-group differences and pairwise comparisons of key biomechanical characteristics for the DS and HS tasks across different FMS score levels.

Task	Features	H value	*p* value	ε^2^	1 vs 2	1 vs 3	2 vs 3
DS	Ankle dorsiflexion angle difference (°)	68.73	<0.001	0.69	<0.001 (r = 0.77)	<0.001 (r = 0.92)	<0.001 (r = −0.95)
	Pelvic vertical displacement ROM (m)	66.40	<0.001	0.66	<0.001 (r = 0.75)	<0.001 (r = 0.93)	<0.001 (r = 0.90)
	Trunk flexion ROM (°)	38.58	<0.001	0.38	<0.001 (r = 0.79)	0.041 (r = 0.37)	<0.001 (r = −0.61)
HS	Hip flexion difference (°)	32.74	<0.001	0.32	<0.001 (r = 0.64)	<0.001 (r = 0.72)	1.000 (r = 0.11)
	Pelvic rotation ROM (°)	26.71	<0.001	0.25	0.578 (r = 0.17)	<0.001 (r = 0.83)	<0.001 (r = −0.93)

Although lumbar spine lateral flexion ROM was identified as a stable feature by machine learning, the univariate Kruskal–Wallis test did not reach statistical significance. For pairwise comparisons, r represents the rank-biserial correlation effect size calculated as $r=1-\frac{2U}{n_{1}n_{2}}$ . A positive r indicates lower values in the first-listed group than in the second-listed group, whereas a negative r indicates higher values in the first-listed group. The absolute value of r reflects the magnitude of the effect. Abbreviations: ROM, range of motion; DS, deep squat; HS, hurdle step.

### 3.3 Machine learning model performance

Table 5 shows the classification performance of different machine learning models for the DS and HS tasks. In the DS task, all models performed well overall, with Random Forest achieving the highest classification performance. Its accuracy, macro F1 score, weighted F1 score, and balanced accuracy were 0.910 ± 0.080, 0.911 ± 0.082, 0.911 ± 0.079, and 0.914 ± 0.085, respectively. The quadratic weighted Kappa was 0.904 ± 0.061, and the MAE was 0.100 ± 0.077. OrdinalRF performed similarly, with an accuracy of 0.900 ± 0.089 and a squared-weighted Kappa of 0.891 ± 0.072. Logistic Regression and SVM achieved accuracies of 0.870 ± 0.068 and 0.880 ± 0.068, respectively, slightly lower than Random Forest. In the HS task, classification performance was generally lower than that observed for the DS task. Among the evaluated models, OrdinalRF achieved the highest descriptive performance, with an accuracy of 0.805 ± 0.053, a macro-F1 score of 0.804 ± 0.054, a weighted F1 score of 0.805 ± 0.052, a balanced accuracy of 0.806 ± 0.054, and a quadratic weighted Kappa of 0.728 ± 0.094. Logistic Regression achieved an accuracy of 0.820 ± 0.010, with a macro-F1 score of 0.817 ± 0.010 and a weighted F1 score of 0.820 ± 0.009. SVM and Random Forest achieved accuracies of 0.780 ± 0.029 and 0.790 ± 0.025, respectively. These results indicate that classification of HS scores remained more challenging than DS scores, with lower overall model performance across algorithms. SVM showed relatively lower performance, with an accuracy of 0.780 ± 0.029. No statistical significance tests were conducted for performance differences between models; therefore, the above results are presented as descriptive comparisons only. Overall, all models achieved higher classification performance in the DS task than in the HS task, suggesting that the key biomechanical features extracted for DS possess better discriminative ability for FMS score levels.

**TABLE 5:** Machine learning model performance.

Task	Model	Accuracy	F1 score	Weighted F1 score	Balanced accuracy	Quadratic weighted kappa	MAE
DS	Logistic	0.870 ± 0.068	0.864 ± 0.069	0.870 ± 0.066	0.869 ± 0.074	0.865 ± 0.059	0.140 ± 0.066
	SVM	0.880 ± 0.068	0.875 ± 0.071	0.878 ± 0.068	0.883 ± 0.075	0.854 ± 0.088	0.140 ± 0.086
	Random forest	0.910 ± 0.080	0.911 ± 0.082	0.911 ± 0.079	0.914 ± 0.085	0.904 ± 0.061	0.100 ± 0.077
	OrdinalRF	0.900 ± 0.089	0.900 ± 0.091	0.900 ± 0.088	0.900 ± 0.091	0.891 ± 0.072	0.110 ± 0.086
HS	Logistic	0.820 ± 0.010	0.817 ± 0.010	0.820 ± 0.009	0.826 ± 0.013	0.698 ± 0.032	0.245 ± 0.019
	SVM	0.780 ± 0.029	0.777 ± 0.031	0.781 ± 0.029	0.789 ± 0.031	0.625 ± 0.066	0.305 ± 0.040
	Random forest	0.790 ± 0.025	0.789 ± 0.025	0.791 ± 0.025	0.797 ± 0.029	0.690 ± 0.062	0.270 ± 0.046
	OrdinalRF	0.805 ± 0.053	0.804 ± 0.054	0.805 ± 0.052	0.806 ± 0.054	0.728 ± 0.094	0.240 ± 0.073

Abbreviations: ROM, range of motion; DS, deep squat; HS, hurdle step.

### 3.4 Model interpretation and analysis

The confusion matrix for the DS task (Figure 5A) shows that the predictions of the Random Forest model were primarily concentrated along the main diagonal. For samples rated 1, 2, and 3, 27/30, 35/40, and 29/30 cases were correctly classified, corresponding to recall rates of 90.0%, 87.5%, and 96.7%, respectively, with an overall accuracy of 91.0%. Misclassifications occurred mainly between adjacent score levels, with only a small number of non-adjacent errors. For the HS task (Figure 5B), the OrdinalRF predictions also showed a predominantly diagonal distribution. For samples rated 1, 2, and 3, 46/60, 65/80, and 50/60 cases were correctly classified, corresponding to recall rates of 76.7%, 81.3%, and 83.3%, respectively, with an overall accuracy of 80.5%. Most HS misclassifications also occurred between adjacent score levels. Compared with the DS task, the HS task showed more off-diagonal errors and lower overall classification accuracy, indicating greater difficulty in distinguishing among HS score categories.

**FIGURE 5:**
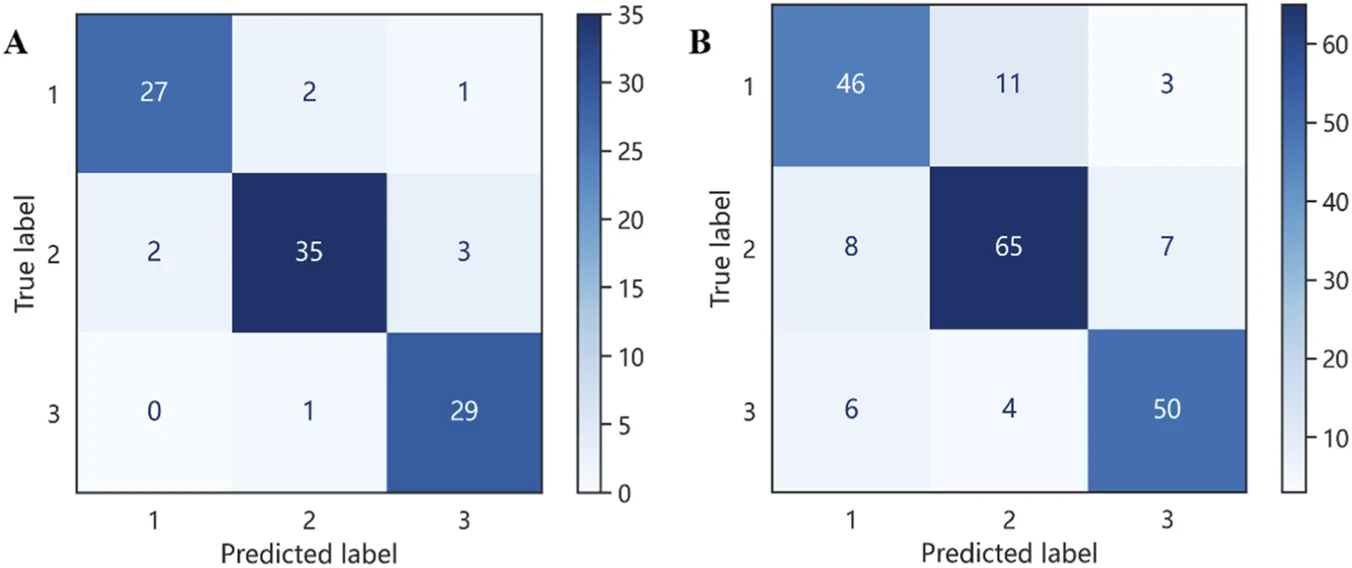
Confusion matrices for the optimal models of the DS and HS tasks. **(A)** Random Forest model for the DS task, based on 100 DS action trials; **(B)** OrdinalRF model for the HS task, based on 200 unilateral HS action trials from 100 participants. The confusion matrices were generated by aggregating the out-of-fold predictions from all five outer cross-validation folds, such that each observation contributed one prediction obtained when it was held out in an outer test fold. Abbreviations: DS, deep squat; HS, hurdle step.

To provide a consistent interpretation of predictive feature relevance across tasks, impurity-based Random Forest feature importance was calculated for both the DS and HS tasks. For the DS task (Figure 6A), ankle dorsiflexion angle difference, pelvic vertical displacement ROM, and trunk flexion ROM showed the highest mean importance values of 0.41, 0.34, and 0.20, respectively. Together, these features accounted for approximately 0.95 of the total Random Forest importance, with the remaining importance distributed across other features not shown in the figure. For the HS task (Figure 6B), hip flexion difference and pelvic rotation ROM showed the highest mean importance values of 0.39 and 0.36, followed by lumbar lateral flexion ROM (0.13). These three features together accounted for approximately 0.88 of the total importance. These importance values indicate predictive relevance within the fitted Random Forest models and should not be interpreted as causal biomechanical effects. Detailed results for features with non-zero importance are provided in Supplementary Table S5.

**FIGURE 6:**
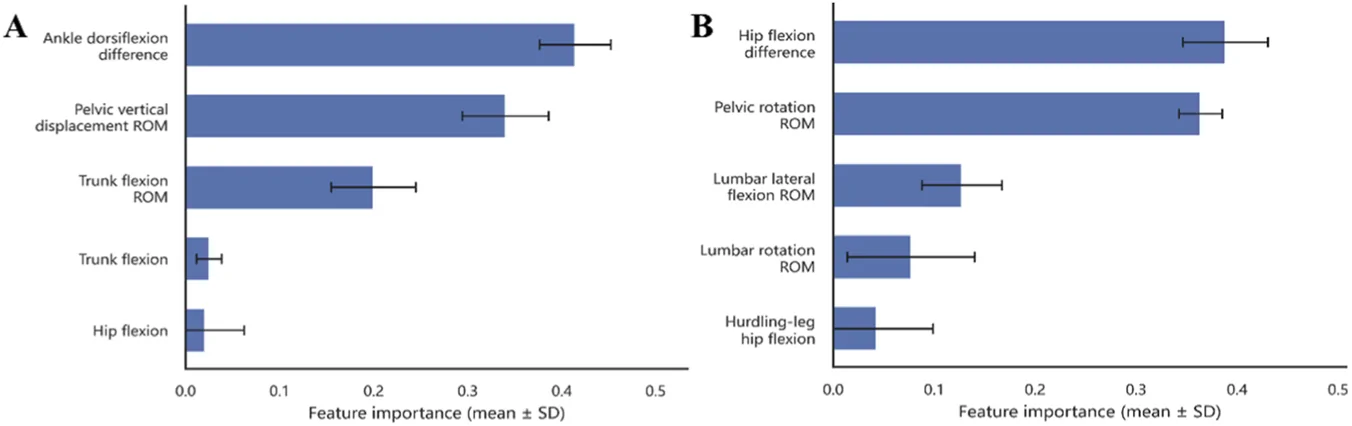
Impurity-based Random Forest feature importance for the DS and HS tasks. **(A)** Feature importance rankings for joint angles and torque metrics related to the squat movement; **(B)** Feature importance rankings for joint angles and pelvic motion metrics related to the hurdle step movement.

## 4 Discussion

### 4.1 Biomechanical characteristics associated with FMS score differences across tasks

The results of this study indicate that the DS and HS tasks exhibit distinct key biomechanical discriminatory features that discriminate across FMS score levels. In the DS task, significant differences across score levels were observed for ankle dorsiflexion angle difference, pelvic vertical displacement ROM, and trunk flexion ROM, suggesting that DS scores are primarily related to lower limb movement symmetry, squat depth, and trunk posture control. As a typical bilateral closed-chain movement, the DS requires multi-joint coordination of the hip, knee, and ankle to perform the squat and maintain postural stability (Cook et al., 2014; Kritz et al., 2009). Previous studies have shown that squat movement quality is closely related to lower limb joint ROM, trunk posture, and lower limb kinematics (Macmillan et al., 2023; Butler et al., 2010). Limited ankle dorsiflexion or bilateral asymmetry in dorsiflexion has been associated with differences in squat depth and compensatory movement patterns, including trunk forward lean and changes in pelvic position. Pelvic vertical displacement ROM reflects, to a certain extent, the squat amplitude achieved by the subject, and trunk flexion ROM reflects trunk posture control and compensation during the squat. Therefore, these features appear to represent biomechanical characteristics associated with differences in DS score levels and showed discriminatory relevance in the present analysis. However, the present DS feature set did not directly quantify several frontal- or transverse-plane compensations relevant to FMS scoring, such as dynamic knee valgus and heel-lift behavior. Their omission may limit the comprehensiveness of biomechanical interpretation and the potential discriminatory information available to the classification models.

In the HS task, hip flexion difference and pelvic rotation ROM demonstrated strong discriminatory power across score levels, indicating that these variables were associated with HS score levels and may characterize aspects of hip coordination and pelvic control under single-leg support conditions. Unlike the DS, the HS is a unilateral dynamic task requiring participants to perform a step-over movement while standing on one leg, which places higher demands on pelvic stability, hip flexion control, and trunk-pelvis coordination (Cook et al., 2014). An increased hip flexion difference may reflect poor coordination between the hurdling leg and the supporting leg, whereas an increased pelvic rotation ROM may indicate compensatory pelvic rotation to complete the hurdling movement. Although lumbar spine lateral flexion ROM was consistently retained during feature selection, no significant differences were observed among the three FMS score groups in the univariate analysis. This indicates that its independent discriminatory ability is limited and that its contribution is more likely to emerge in combination with other biomechanical features within the multivariable classification model. Therefore, in the present dataset, hip flexion difference and pelvic rotation control showed stronger statistical and discriminatory associations with HS score levels than lumbar lateral flexion ROM. Notably, the HS candidate feature set also included frontal- and transverse-plane measures, including pelvic lateral tilt, pelvic rotation, and lumbar lateral flexion and rotation, although not all were retained as stable discriminatory features. It should be noted that the HS statistical comparisons and machine learning analyses were conducted at different analytical levels. Therefore, concordance between statistically significant features and model feature importance should not be interpreted as a direct one-to-one correspondence, but rather as complementary evidence obtained from participant-level and unilateral-trial-level analyses.

Existing studies have primarily focused on sagittal plane kinematic characteristics in the DS task (e.g., trunk, hip, knee, ankle flexion), while relatively little attention has been paid to unilateral support and pelvic control characteristics in the HS task (Macmillan et al., 2023; Butler et al., 2010). By comparing the DS and HS tasks within the same analytical framework, the present findings indicate task-specific associations between FMS score levels and biomechanical characteristics. In the present dataset, DS score levels were more strongly associated with ankle symmetry, squat depth, and trunk posture variables, whereas HS score levels were more strongly associated with hip coordination and pelvic rotation variables.

The machine learning results further support the aforementioned task differences. In the DS task, Random Forest showed the highest descriptive classification performance, suggesting that the identified biomechanical features provided relatively strong discrimination among DS score levels. In contrast, classification performance was lower in the HS task, with OrdinalRF showing the highest descriptive performance among the evaluated models. However, the overall performance remained lower than that observed for the DS task, indicating greater difficulty in distinguishing among HS score levels. Misclassifications mainly occurred between adjacent score categories, suggesting that the biomechanical boundaries between neighboring HS scores may be less distinct. Previous studies have also reported performance differences across movement tasks in automatic FMS scoring based on kinematic data, IMU sensors, or machine learning methods exhibits (Remedios et al., 2020; Shen et al., 2024; Xing et al., 2022; Wu et al., 2020). As a unilateral dynamic task, HS movement quality is influenced by multiple factors, including pelvic, hip, and trunk control, as well as side-specific differences, which may result in more ambiguous grading boundaries. Therefore, when constructing an objective FMS scoring model, the DS task may be classified more stably using a small set of key features, whereas the HS task may require integrating multi-dimensional movement control information to further enhance the model’s discriminatory power.

### 4.2 Causes of confusion between adjacent rating categories: the combined effects of the rating system and biomechanics

This study found that model misclassifications primarily occurred between adjacent rating categories, with the distinction between a score of 2 and adjacent categories proving particularly challenging. This phenomenon may stem from both the discrete nature of the FMS scoring system and the continuous nature of biomechanical performance across score levels. FMS scoring relies primarily on the evaluator’s observational judgment of movement quality, which involves a degree of subjectivity; thus, differences in interpretation and judgment may exist among different evaluators (Moran et al., 2016; Leeder et al., 2016). At the same time, the FMS scoring system categorizes continuous movement quality into 1-, 2-, and 3-point levels, but actual movement performance is not strictly separated. Different particilants may complete the same movement using different compensatory strategies, leading to some overlap between score levels.

In the DS task, significant differences in ankle dorsiflexion angle difference, pelvic vertical displacement ROM, and trunk flexion ROM were observed across most scoring levels, indicating that DS scoring levels exhibit a clear biomechanical gradient. This may explain why model classification performance is relatively better for DS task and why the confusion matrix shows a more clearly clustered main diagonal. In contrast, for the HS task, hip flexion difference primarily distinguishes the 1-point group from the higher-scoring groups, while the difference between the 2-point and 3-point groups is not significant. Pelvic rotation ROM shows differences primarily between the 1-point and 3-point groups, as well as between the 2-point and 3-point groups. This suggests that the intermediate score levels in the HS task do not simply lie between low and high scores, but may represent an intermediate state in which the movement is performed through pelvic rotation or hip joint compensation. Therefore, feature overlap and model misclassification are more likely to occur between adjacent score levels in the HS task.

Furthermore, inter-rater reliability may also influence the model’s learning of rating scale boundaries. In this study, the weighted kappa coefficients for the DS and HS tasks were 0.90 and 0.66, respectively, indicating lower inter-rater reliability for the HS task than for the DS task. Previous studies have shown that inter-rater reliability in FMS scoring is influenced by factors such as rater experience, training level, and movement task complexity (Moran et al., 2016; Leeder et al., 2016). Because machine learning models use human FMS scores as training labels, the inherent uncertainty in these labels may further increase the difficulty of distinguishing between adjacent grades and constrain the achievable model performance, particularly for the HS task. Therefore, the model should be interpreted as approximating evaluator-assigned FMS scores rather than establishing an independent gold standard of movement quality. Future efforts to establish objective FMS scoring models could further integrate methods such as expert consensus scoring, repeated scoring, or multi-rater label fusion improve label quality and reduce confusion between adjacent grades.

It should also be noted that the present study used a stratified purposive sampling strategy to obtain relatively balanced score categories for model training and comparison. This design was useful for examining biomechanical differences across FMS score levels and improving model stability under small-sample conditions. However, in real-world screening settings, FMS scores are often naturally imbalanced, with some score categories occurring more frequently than others. Accordingly, the classification accuracy reported in the present study should be interpreted as internal performance within the deliberately constructed analytical sample rather than as an estimate of performance in real-world screening populations. Therefore, the classification performance observed in this study may be higher than that achieved in practical populations with uneven score distributions. Future studies should further evaluate the model using continuously recruited samples or external validation cohorts to determine its real-world generalizability.

## 5 Limitations

This study has certain limitations. First, the sample size was relatively small, and all participants were healthy adolescents from physical education colleges. Moreover, the use of stratified purposive sampling resulted in a score distribution that differed from the natural distribution expected in real-world screening populations. Therefore, the reported classification performance should be interpreted as internal performance within this deliberately constructed analytical sample and should not be directly generalized to real-world screening settings. Future studies should include larger and more diverse samples with naturally occurring FMS score distributions and independent external validation cohorts. Second, although several key biomechanical features were identified using 3D motion capture and inverse dynamics analysis, these variables mainly describe external movement patterns and joint mechanics and cannot fully explain underlying neuromuscular mechanisms. In addition, some relevant frontal- and transverse-plane compensations, particularly dynamic knee valgus and heel lift in the DS task, were not directly quantified. Future studies should therefore incorporate additional multi-planar biomechanical measures together with multimodal data, such as surface electromyography and plantar pressure measurements, to provide a more comprehensive characterization of movement quality. Third, the machine learning models were trained using manual FMS scores as reference labels. Although inter-rater agreement was acceptable, the lower reliability observed for the HS task may have introduced label uncertainty and constrained model performance, especially between adjacent score levels. For the HS task, the use of participant-level statistical analysis and unilateral-trial-level machine learning may limit direct comparison between statistical results and model feature importance. Fourth, each movement was analyzed using a single valid trial, which was consistent with the standardized FMS assessment procedure but may not fully capture within-subject movement variability. Although standardized instructions and quality-control procedures were applied, repeated trials may provide additional information regarding movement consistency and reliability. Future studies should investigate test–retest reliability and incorporate multiple trials to better characterize individual movement variability.

Finally, although subject-level aggregation and GroupKFold cross-validation were used to reduce the influence of within-subject correlation in the HS task, future studies could further apply mixed-effects or hierarchical models to better account for side-specific differences.

## 6 Conclusion

This study integrated 3D motion capture, inverse kinematics, and machine learning to identify distinct biomechanical discriminators for FMS deep squat and hurdle step tasks. DS scores were primarily associated with ankle dorsiflexion asymmetry, pelvic vertical displacement ROM, and trunk flexion ROM, whereas HS scores with hip flexion difference and pelvic rotation ROM. Lumbar spine lateral flexion ROM may serve as auxiliary discriminatory information in a multi-feature model. These findings indicate that different FMS movement tasks may be associated with distinct biomechanical discriminatory characteristics. The identified biomechanical features may provide quantitative information that could inform the future development of individualized training or rehabilitation approaches; however, their applicability for guiding such interventions requires further validation in prospective intervention studies.

## Data Availability

The raw data supporting the conclusions of this article will be made available by the authors, without undue reservation.
