# Supplementary material for: Objective classification of deep squat and hurdle step scores in functional movement screen: a 3D motion capture and machine learning approach
Source: Front Bioeng Biotechnol. 2026 Sep 22;14:1898662. doi: 10.3389/fbioe.2026.1898662 (PMC13638601; doi:10.3389/fbioe.2026.1898662)
Supplement: Supplementary file 1 [file Table1.docx]

**Supplementary Table S1. Anatomical locations of the 36 experimental reflective markers and their correspondence to the customized OpenSim marker set.**

| **No.** | **Experimental marker** | **Anatomical location** | **Side** | **Corresponding OpenSim virtual marker** |
| --- | --- | --- | --- | --- |
| **1** | **C7** | **Posterior base of the neck (spinous process of C7)** | **Midline** | **C7** |
| **2** | **T10** | **Posterior trunk at the T10 level** | **Midline** | **T10** |
| **3** | **CLAV** | **Clavicle** | **Midline** | **CLAV** |
| **4** | **STRN** | **Midpoint of the upper-body midline on the sternum** | **Midline** | **STRN** |
| **5** | **LShoulderFront** | **Anterior aspect of the left shoulder** | **Left** | **LShoulderFront** |
| **6** | **LShoulderBack** | **Posterior aspect of the left shoulder** | **Left** | **LShoulderBack** |
| **7** | **LElbow** | **Lateral epicondyle of the left humerus** | **Left** | **LElbow** |
| **8** | **LElbowIn** | **Medial epicondyle of the left humerus** | **Left** | **LElbowIn** |
| **9** | **LWristIn** | **Radial styloid process of the left wrist** | **Left** | **LWristIn** |
| **10** | **LWristOut** | **Ulnar styloid process of the left wrist** | **Left** | **LWristOut** |
| **11** | **LHandIn** | **Base of the second finger joint of the left hand** | **Left** | **LHandIn** |
| **12** | **LHandOut** | **Fifth finger joint region of the left hand (approximately 2 cm below the joint base)** | **Left** | **LHandOut** |
| **13** | **RShoulderFront** | **Anterior aspect of the right shoulder** | **Right** | **RShoulderFront** |
| **14** | **RShoulderBack** | **Posterior aspect of the right shoulder** | **Right** | **RShoulderBack** |
| **15** | **RElbow** | **Lateral epicondyle of the right humerus** | **Right** | **RElbow** |
| **16** | **RElbowIn** | **Medial epicondyle of the right humerus** | **Right** | **RElbowIn** |
| **17** | **RWristIn** | **Radial styloid process of the right wrist** | **Right** | **RWristIn** |
| **18** | **RWristOut** | **Ulnar styloid process of the right wrist** | **Right** | **RWristOut** |
| **19** | **RHandIn** | **Base of the second finger joint of the right hand** | **Right** | **RHandIn** |
| **20** | **RHandOut** | **Fifth finger joint region of the right hand (approximately 2 cm below the joint base)** | **Right** | **RHandOut** |
| **21** | **LASIS** | **Left anterior superior iliac spine** | **Left** | **LASIS** |
| **22** | **LPSIS** | **Left posterior superior iliac spine** | **Left** | **LPSIS** |
| **23** | **RASIS** | **Right anterior superior iliac spine** | **Right** | **RASIS** |
| **24** | **RPSIS** | **Right posterior superior iliac spine** | **Right** | **RPSIS** |
| **25** | **LKness** | **Lateral femoral condyle of the left knee** | **Left** | **LKness** |
| **26** | **LKnessIn** | **Medial femoral condyle of the left knee** | **Left** | **LKnessIn** |
| **27** | **LAnkleOut** | **Lateral malleolus of the left ankle** | **Left** | **LAnkleOut** |
| **28** | **LHeel** | **Posterior heel of the left foot** | **Left** | **LHeel** |
| **29** | **LMT5** | **Lateral aspect of the left forefoot (fifth metatarsal region)** | **Left** | **LMT5** |
| **30** | **LMT1** | **Medial aspect of the left forefoot (first metatarsal region)** | **Left** | **LMT1** |
| **31** | **RKness** | **Lateral femoral condyle of the right knee** | **Right** | **RKness** |
| **32** | **RKnessIn** | **Medial femoral condyle of the right knee** | **Right** | **RKnessIn** |
| **33** | **RAnkleOut** | **Lateral malleolus of the right ankle** | **Right** | **RAnkleOut** |
| **34** | **RHeel** | **Posterior heel of the right foot** | **Right** | **RHeel** |
| **35** | **RMT5** | **Lateral aspect of the right forefoot (fifth metatarsal region)** | **Right** | **RMT5** |
| **36** | **RMT1** | **Medial aspect of the right forefoot (first metatarsal region)** | **Right** | **RMT1** |

**Note: The experimental marker set was reproduced as a customized 36-marker virtual marker set in the OpenSim model, with each experimental marker mapped to the corresponding anatomical landmark. Marker names shown in the final column refer to the customized virtual marker names used for correspondence with the experimental trajectories.**

**Table S2 List of all features extracted for the DS and HS tasks**

| **Deep Squat（DS, n = 14）** | | | |
| --- | --- | --- | --- |
| **Category** | **Feature Name** | **Definition** | **Calculation Method** |
| Lower Limb Kinesiology | Hip Flexion Angle | Representative peak values of hip flexion angle during the squat | The maximum value in the time series of hip flexion angle |
|  | Knee Flexion Angle | Representative peak values of knee flexion angle during squats | The maximum value in the time series of knee flexion angles |
|  | Ankle Dorsiflexion Angle | Representative peak values of ankle dorsiflexion during squats | The maximum value in the time series of ankle dorsiflexion angles |
| Trunk posture | Trunk Flexion Angle | Representative peak values of trunk flexion angle during squats | The maximum value in the time series of trunk flexion angle |
|  | Trunk Flexion ROM | Range of trunk flexion during a squat | Maximum − Minimum |
| Trunk-Lower Limb Coordination | Trunk-Lower Leg Angle Difference | Differences in relative posture between the trunk and lower leg segments | Calculate the absolute value of the difference between the trunk angle and the lower leg angle |
| Symmetry | Difference in Knee Flexion Angle | Difference in knee flexion angles between the left and right knees | The absolute value of the difference between the peak knee flexion angles on the left and right sides |
|  | Ankle Dorsiflexion Angle Difference | Ankle Dorsiflexion Angle Difference s between the left and right sides | Absolute value of the difference between the peak ankle dorsiflexion angles on the left and right sides |
| Pelvic Control | ROM of Pelvic Anterior/Posterior Tilt | Range of pelvic tilt during a squat | Maximum − Minimum |
|  | ROM of Pelvic Vertical Displacement | The range of vertical displacement of the pelvis during a squat, reflecting the depth of the squat | Maximum − Minimum |
|  | Peak Pelvic Vertical Position | Representative peak values of pelvic vertical position during the squat | Find the peak in the time series of the vertical position of the pelvis |
| Kinetics | Peak Hip Joint Torque | Peak hip joint torque during the squat | Take the peak value of the hip joint torque time series; take the absolute value if necessary |
|  | Peak Knee Joint Torque | Peak knee joint torque during squats | Take the peak value of the knee joint torque time series; take the absolute value if necessary |
|  | Peak Ankle Joint Torque | Peak ankle joint torque during the squat | Take the peak value of the ankle joint torque time series; take the absolute value if necessary |
| **Hurdle Step（HS，n = 15）** | | | |
| **Category** | **Feature Name** | **Definition** | **Calculation Method** |
| Kinematics of the Hurdling Leg | Hips Flexion Angle of Hurdling Leg | Representative peak values of hip flexion angle in the hurdling leg during the hurdling stride | Find the maximum value of the hip flexion angle during the hurdle leg in the time series |
|  | Ankle Angle of Hurdling Leg | Representative Peak Values of the Ankle Angle of the Hurdling Leg During the Hurdling Stride | Take the maximum value of the time series of the ankle angle of the hurdling leg |
|  | Knee Flexion Angle of Support Leg | Representative peak values of the supporting leg knee flexion angle during the hurdle stride | The maximum value in the time series of the supporting leg knee flexion angle |
| Trunk posture | Trunk Lateral Flexion | Angle of trunk lateral flexion in the frontal plane during the hurdle stride | Extract representative peak values from the time series of trunk lateral flexion angles |
|  | Trunk Forward Flexion | Angle of trunk flexion in the sagittal plane during the hurdle stride | Extract representative peak value of the time series for the trunk flexion angle |
| Symmetry | Hip Flexion Difference | Difference in hip flexion angles between the stride leg and the support leg | Absolute value of the difference between the peak hip flexion angles on both sides |
| Pelvic Control | Pelvic Lateral Tilt | Angle of pelvic lateral tilt in the frontal plane during the hurdling stride | Extract representative peak values from the time series of pelvic lateral tilt angles |
|  | Pelvic Rotation ROM | Range of pelvic rotation during the hurdle stride | Maximum − Minimum |
| Lumbar Spine Kinesiology | Lumbar Spine Forward Flexion ROM | Range of lumbar flexion during the hurdle stride | Maximum − Minimum |
|  | Lumbar Spine Lateral Flexion ROM | Range of lumbar lateral flexion angles during the hurdle stride | Maximum value − Minimum value |
|  | Lumbar Spine Rotation ROM | Range of lumbar spine rotation during the hurdle stride | Maximum value − Minimum value |
| Kinetics | Peak Hip Joint Torque of The Hurdling Leg | Peak hip joint torque of the hurdling leg during the hurdle stride | Take the peak value from the time series of hip joint torque of the hurdling leg; take the absolute value if necessary |
|  | Peak Hip Joint Torque of The Supporting Leg | Peak hip joint torque of the supporting leg during the hurdle stride | Take the peak value from the time series of hip joint torque of the supporting leg; take the absolute value if necessary |
|  | Peak Knee Joint Torque of The Supporting Leg | Peak knee joint torque of the supporting leg during the hurdle stride | Take the peak value of the time series of the supporting leg’s knee joint torque; take the absolute value if necessary |
|  | Peak Ankle Joint Torque of The Hurdling Leg | Peak value of the hurdling leg’s ankle joint torque during the hurdle stride | Take the peak value of the time series of the hurdling leg’s ankle joint torque; take the absolute value if necessary |

**Table S3. Class distributions before and after random oversampling in the five outer-training folds.**

| **Task** | **Outer Fold** | **Before ROS: Score 1** | **Score 2** | **Score 3** | **After ROS: Score 1** | **Score 2** | **Score 3** |
| --- | --- | --- | --- | --- | --- | --- | --- |
| DS | 1 | 24 | 30 | 26 | 30 | 30 | 30 |
| DS | 2 | 23 | 34 | 23 | 34 | 34 | 34 |
| DS | 3 | 26 | 30 | 24 | 30 | 30 | 30 |
| DS | 4 | 22 | 32 | 26 | 32 | 32 | 32 |
| DS | 5 | 25 | 34 | 21 | 34 | 34 | 34 |
| HS | 1 | 46 | 67 | 47 | 67 | 67 | 67 |
| HS | 2 | 49 | 65 | 46 | 65 | 65 | 65 |
| HS | 3 | 50 | 60 | 50 | 60 | 60 | 60 |
| HS | 4 | 47 | 64 | 49 | 64 | 64 | 64 |
| HS | 5 | 48 | 64 | 48 | 64 | 64 | 64 |

Note: RandomOverSampler with sampling_strategy = "auto" was applied only to the training data. Minority classes were randomly oversampled with replacement until each class matched the sample size of the largest class within the corresponding training partition.

**Table S4 Results of Tests for Normality and Homoscedasticity of Features in the DS and HS Tasks**

| **Task** | **Feature Name** | **Shapiro-Wilk *p* value：1 score** | **Shapiro-Wilk *p* value：2 score** | **Shapiro-Wilk *p* value：3 score** | **Levene *p* value** |
| --- | --- | --- | --- | --- | --- |
| DS | Hip Flexion Angle | <0.001 | <0.001 | <0.001 | 0.247 |
|  | Knee Flexion Angle | 0.191 | 0.439 | 0.192 | 0.998 |
|  | Ankle Dorsiflexion Angle | 0.292 | 0.016 | 0.627 | 0.738 |
|  | Trunk Flexion Angle | <0.001 | <0.001 | <0.001 | 0.312 |
|  | Trunk-Lower Leg Angle Difference | <0.001 | <0.001 | 0.005 | 0.989 |
|  | Difference in Knee Flexion Angle | 0.008 | 0.018 | 0.030 | 0.644 |
|  | Ankle Dorsiflexion Angle Difference | 0.002 | 0.008 | 0.124 | 0.002 |
|  | ROM of Pelvic Anterior/Posterior Tilt | 0.451 | 0.331 | 0.922 | 0.521 |
|  | Trunk flexion ROM | 0.020 | 0.004 | <0.001 | 0.231 |
|  | ROM of Pelvic Vertical Displacement | <0.001 | 0.151 | 0.047 | 0.463 |
|  | Peak Pelvic Vertical Position | 0.539 | 0.086 | 0.046 | 0.468 |
|  | Peak Hip Joint Torque | <0.001 | <0.001 | 0.003 | 0.632 |
|  | Peak Knee Joint Torque | <0.001 | <0.001 | <0.001 | 0.820 |
|  | Peak Ankle Joint Torque | <0.001 | <0.001 | 0.031 | 0.626 |
| HS | Hips Flexion Angle of Hurdling Leg | 0.483 | 0.173 | 0.443 | 0.800 |
|  | Knee Flexion Angle of Support Leg | 0.037 | 0.378 | 0.580 | 0.062 |
|  | Ankle Angle of Hurdling Leg | 0.278 | 0.006 | 0.610 | 0.006 |
|  | Trunk Lateral Flexion | <0.001 | <0.001 | <0.001 | 0.085 |
|  | Trunk Forward Flexion | <0.001 | <0.001 | <0.001 | 0.257 |
|  | Pelvic Lateral Tilt | 0.010 | 0.861 | 0.992 | 0.164 |
|  | Hip Flexion Difference | 0.359 | 0.732 | 0.804 | 0.871 |
|  | Pelvic Rotation ROM | 0.004 | 0.163 | 0.052 | 0.020 |
|  | Lumbar Spine Forward Flexion ROM | <0.001 | <0.001 | <0.001 | 0.534 |
|  | Lumbar Spine Lateral Flexion ROM | <0.001 | <0.001 | <0.001 | 0.511 |
|  | Lumbar Spine Rotation ROM | <0.001 | <0.001 | <0.001 | 0.477 |
|  | Peak Hip Joint Torque of The Hurdling Leg | <0.001 | <0.001 | <0.001 | 0.536 |
|  | Peak Hip Joint Torque of The Supporting Leg | <0.001 | <0.001 | <0.001 | 0.764 |
|  | Peak Knee Joint Torque of The Supporting Leg | <0.001 | <0.001 | 0.012 | 0.389 |
|  | Peak Ankle Joint Torque of The Hurdling Leg | <0.001 | <0.001 | <0.001 | 0.846 |

Note: Statistical analysis for the DS task was conducted at the trial level, with sample sizes for each rating category as follows: 1 point = 30, 2 points = 40, and 3 points = 30. Statistical analysis for the HS task was conducted at the participant level; the mean of the continuous biomechanical features from the left and right sides of the same participant was calculated, and the participant’s HS score was determined based on the lower score between the left and right sides; After aggregation, the sample sizes for each rating level are 1 point = 52, 2 points = 35, and 3 points = 13. Normality within each rating level was assessed using the Shapiro-Wilk test, and homogeneity of variances across the three rating levels was assessed using the Levene test. If the p-value of the Shapiro-Wilk test for any score category was < 0.05, the feature was deemed non-normal; if the p-value of the Levene test was < 0.05, the variances were deemed unequal. P-values less than 0.001 were uniformly reported as < 0.001.

**Table S5 Feature importance results for non-zero contributing features in the Random Forest models of the DS and HS tasks**

| **Task** | **Ranking** | **Feature Name** | **Importance (mean ± SD)** | **Cumulative importance** | **Residual importance** |
| --- | --- | --- | --- | --- | --- |
| DS | 1 | Ankle Dorsiflexion Angle Difference | 0.414 ± 0.038 | 0.414 | 0.586 |
|  | 2 | Pelvic Vertical Displacement ROM | 0.340 ± 0.046 | 0.754 | 0.246 |
|  | 3 | Trunk Flexion ROM | 0.200 ± 0.045 | 0.954 | 0.046 |
|  | 4 | Trunk Flexion Angle | 0.025 ± 0.013 | 0.979 | 0.021 |
|  | 5 | Hip Flexion Angle | 0.021 ± 0.041 | 1.000 | 0.000 |
| HS | 1 | Hip Flexion Difference | 0.388 ± 0.042 | 0.388 | 0.612 |
|  | 2 | Pelvic Rotation ROM | 0.364 ± 0.021 | 0.752 | 0.248 |
|  | 3 | Lumbar Spine Lateral Flexion ROM | 0.128 ± 0.040 | 0.880 | 0.120 |
|  | 4 | Lumbar Spine Rotation ROM | 0.077 ± 0.063 | 0.957 | 0.043 |
|  | 5 | Hips Flexion Angle of Hurdling Leg | 0.043 ± 0.056 | 1.000 | 0.000 |

Note: Feature importance was calculated from the Random Forest model using 5-fold GroupKFold cross-validation. Values are presented as the mean ± standard deviation across the five folds. Cumulative importance was calculated after sorting features in descending order of mean importance, and residual importance was defined as 1 minus cumulative importance. Only features with non-zero mean importance are shown; features with an average importance of 0.000 ± 0.000 were omitted from the table. Figure 6 presents only the top-ranked features, whereas this table provides the detailed results for all non-zero contributing features.
